# Optimal treatment of patients with NSTE-ACS in the Dutch health care system

**DOI:** 10.1007/s12471-018-1114-4

**Published:** 2018-04-30

**Authors:** J. P. van Kuijk, J. M. ten Berg

**Affiliations:** Department of Cardiology, St Antonius Hospital, Nieuwegein, The Netherlands

**Keywords:** therapeutics, acute coronary syndrome, myocardial infarction

## Abstract

In the current daily practice of acute coronary syndromes, patients experiencing non-ST-elevation acute coronary syndrome (NSTE-ACS) represent the majority of this population. In these patients it is of utmost importance to estimate both ischaemic and bleeding risk, with subsequent, and preferably tailored pharmacological and, if indicated, invasive treatment. In this paper we describe the several risk scores and evaluate which are most applicable to the Dutch health-care system. Furthermore, we provide an overview of the recommended pharmacological treatment in keeping with the European Society of Cardiology guidelines. An important topic of this paper is how to decide between early or delayed invasive strategies. We describe the recommendations of the European Society of Cardiology and evaluate to which level these should be applied to the Dutch health-care system.

## Introduction

Non-ST-elevation acute coronary syndromes (NSTE-ACS) comprise a wide clinical spectrum, ranging from patients free of symptoms at presentation to those with ongoing ischaemia, haemodynamic or electrical instability or even cardiac arrest. In patients with unstable angina, there is myocardial ischaemia without cell loss, however in those with non-ST-elevation myocardial infarction (NSTEMI), there is cardiomyocyte necrosis, characterised by elevated troponin and/or creatine kinase levels.

Patients without an ST-elevation myocardial infarction (STEMI) and no haemodynamic or electrical instability, will be presented to cardiac emergency departments where several diagnostic tools should be available. A 12-lead ECG is obtained and interpreted <10 min after arrival [1]. Next, it is mandatory to measure biomarkers of cardiomyocyte injury, preferably high-sensitive troponin, which have increased sensitivity and diagnostic accuracy for the detection of acute myocardial infarction (AMI) [2]. Importantly, the absolute troponin change (delta) within 1 h can be used as a surrogate for absolute changes over the previously used 3–6 h [3]. The cut-off levels within the 0/1 h algorithm depend on the specific assay that is used to measure troponin levels [4, 5]. The specific cut-off values for the most commonly used assays are presented in the 2015 European Society of Cardiology (ESC) guidelines for the management of acute coronary syndromes in patients presenting without persistent ST-segment elevation, [6].

In this paper we will describe the recommendations of the 2015 ESC guidelines on risk stratification and treatment of NSTE-ACS patients. In addition, we will give advice on how to apply these recommendations in daily clinical practice in the Dutch health care system.

**Figure:**
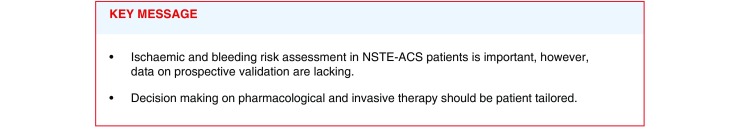

## Risk assessment

Based on the clinical presentation, the initial ECG and the biomarkers, an ischaemic and bleeding risk assessment should be performed in every individual patient, as these assessments provide not only diagnostic but also prognostic information [7–9].

### Ischaemic risk

Quantitative assessment of the ischaemic risk in patients with NSE-ACS, using specific risk scores, is superior to clinical assessment alone. The Global Registry of Acute Coronary Events (GRACE) risk score provides the most accurate risk stratification, whereas the GRACE 2.0 risk calculator provides a direct estimation of in-hospital mortality, at 6 months, at 1 year and at 3 years. In addition, the combined risk of death and myocardial infarction (MI) at 1 year is also provided [10]. Importantly, although the value of risk scores as prognostic assessment tools is undisputed, the impact of using risk scores in daily practice on patient outcomes has not yet been adequately investigated [11]. In NSTE-ACS, the risk is highest at the time of presentation, and therefore, accurate assessment of acute risk guides initial evaluation, selection of the site of care, and therapy [12]. Therapy includes antithrombotic treatment and (timing of) coronary angiography, which will be discussed later in this paper.

In conclusion, the ESC guidelines recommend using established risk scores for prognosis assessment (Class IB) [6, 13]. For the Dutch health care system, we advise using the GRACE risk score for general risk estimation at admission as this is easily accessible and has proven accuracy.

### Bleeding risk

As platelet inhibition/anticoagulation is the cornerstone in the treatment of suspected NSTE-ACS and major bleeding events are associated with increased mortality in NSTE-ACS, assessing bleeding risk is of utmost importance [14]. Two bleeding risk scores are mentioned in the 2015 NSTE-ACS ESC guidelines; CRUSADE (Can Rapid risk stratification of Unstable angina patients Suppress Adverse outcomes with Early implementation of the ACC/AHA guidelines) and ACUITY (Acute Catheterization and Urgent Intervention Triage strategy) [14, 15]. Overall, in ACS patients undergoing coronary angiography, the CRUSADE score was found to be the most discriminatory [16]. In addition, the predictive value of both scores has not been established in patients who are treated medically or in those on oral anticoagulation.

In conclusion, in the ESC guidelines the use of the CRUSADE score has a Class IIb recommendation to quantify bleeding risk in patients undergoing coronary angiography [6]. In our opinion, the CRUSADE score can be used to quantify bleeding risk in patients undergoing coronary angiography, and in patients with an increased risk the use of platelet inhibitors should be patient tailored.

## Pharmacological treatment

The primary goals of the pharmacological treatment are decreasing myocardial oxygen demand and increasing myocardial oxygen supply. Nitrates are primarily used for symptom relief and should be used in patients with ongoing angina. Beta-blockers provide a 13% relative risk reduction of mortality in the first week after myocardial infarction in NSTE-ACS [17]. In the acute phase beta-blockers can be used to lower the heart rate in patients with ischaemia driven by tachycardia. However, early administration of beta-blockers should be avoided in patients if the ventricular function is unknown [18].

### Platelet inhibition

In the pre-PCI era, several randomised trials and meta-analysis have demonstrated the effectiveness of aspirin in patients with unstable angina [19–22]. Several oral, and one intravenous, P2Y12 inhibitors have been developed during the last 2 decades, including clopidogrel, prasugrel, ticagrelor and cangrelor, respectively. The mechanism of action is inhibition of platelet aggregation that is induced by adenosine diphosphate. Clopidogrel and prasugrel irreversibly inactivate the platelet P2Y12 receptors, whereas ticagrelor is a reversible P2Y12 inhibitor with a plasma half-life of 6–12 h. Cangrelor is effective in 2 min after intravenous administration and the duration of effect is 1–2 h, whereas plasma half-life of the active P2Y12 inhibitor is 5–10 min.

#### Clopidogrel

The CURE (Clopidogrel in Unstable Angina to Prevent Recurrent Events) trial was the first study that demonstrated a beneficial effect of adding clopidogrel to aspirin in patients with NSTE-ACS (composite of cardiovascular death, MI or stroke, 9.3% vs. 11.4%; hazard ratio [HR] clopidogrel vs. aspirin 0.80 95% confidence interval [CI] 0.72–0.90), but this was associated with an absolute risk increase of major bleeding of 1% (3.7% vs. 2.7%, relative risk 1.38, *p* = 0.001) [23]. In addition, 12,562 ACS patients treated with this combination of platelet inhibitors, had up to 10% risk of recurrent ischaemic events in the first year, with up to 2% stent thrombosis [24]. It is also worth noting that in this study only 2,658 (21%) patients underwent PCI and 2,072 (17%) coronary artery bypass grafting (CABG), therefore, the majority was treated medically.

#### Prasugrel

The more potent P2Y12 inhibitor prasugrel was tested against clopidogrel in the TRITON-TIMI 38 (TRial to Assess Improvement in Therapeutic Outcomes by Optimizing Platelet InhibitioN with Prasugrel-Thrombolysis in Myocardial Infarction 38) trial, including 10,074 NSTE-ACS patients scheduled for PCI [25]. At 15-month follow-up the composite endpoint was reduced in prasugrel-treated patients (cardiovascular death, non-fatal MI, non-fatal stroke, 9.9% vs. 12.1%; HR prasugrel vs. clopidogrel 0.81, 95% CI 0.73–0.90), driven by a significant reduction in myocardial infarction (24% relative risk reduction), however, this was at the cost of a 40% increase in severe non-CABG-related bleeding events (2.4% vs. 1.8%, *p* = 0.02). Prasugrel is contraindicated in patients with prior stroke/transient ischaemic attack (TIA) due to net evidence of harm in this group. Post-hoc analysis demonstrated no net clinical benefit in patients with a low bodyweight (<60 kg) or those aged >75 years using prasugrel compared with clopidogrel. In these patients prasugrel could be reduced to 5 mg daily or replaced by clopidogrel.

#### Ticagrelor

The most recent oral P2Y12 inhibitor ticagrelor was tested against clopidogrel in the PLATO (PLATelet inhibit and patient Outcomes) trial, including 18,624 patients who had a moderate to high risk of NSTE-ACS (planned for either conservative or invasive management) or STEMI [26]. Ticagrelor demonstrated a 17% relative risk reduction in the primary composite efficacy endpoint (composite of death from cardiovascular causes, MI or stroke, 9.8% vs. 11.7%; HR ticagrelor vs. clopidogrel 0.84 95% CI 0.77–0.92). Ticagrelor was associated with an 28% increased risk of non-CABG-related PLATO-defined major bleeding events (4.5% vs. 3.8%, *p* = 0.03), but no difference in life-threatening or fatal bleeding events. Important contraindications include active pathological bleeding, history of intracranial haemorrhage and severe hepatic impairment. In addition, dyspnoea is relatively often reported with ticagrelor use and caution should be taken in patients at risk for bradycardia events. Finally, ticagrelor has an increased risk of interaction with other CYP3A4 inhibitors.

#### Cangrelor

The intravenous adenosine triphosphate (ATP) analogue cangrelor has a high affinity for the P2Y12 receptor but binds reversibly and has a short plasma half-life (<10 min). The CHAMPION trials have tested cangrelor, and a meta-analysis of these trials observed a 19% relative risk reduction in periprocedural death, MI, ischaemia-driven revascularisation and stent thrombosis, compared with clopidogrel [27]. Thrombolysis in myocardial infarction (TIMI) major and minor bleeding rates were increased, but no increase in the transfusion rate. In 2015, the European Commission issued marketing authorisation for this compound. Important contra-indications include active or increased risk of bleeding and any history of stroke/TIA.

The ESC Task Force for the management of ACS in patients presenting with NSTE-ACS recommended initiating P2Y12 inhibitors soon after the diagnosis is established [28]. In daily practice, this implies pre-treatment with P2Y12 inhibitors in patients who are scheduled for an invasive approach. Currently, the only randomised trial investigating pre-treatment with P2Y12 inhibitors was the ACCOAST trial (Comparison of Prasugrel at the Time of Percutaneous Coronary Intervention or as Pretreatment at the Time of Diagnosis in Patients with Non-ST Elevation Myocardial Infarction trial) [29]. TIMI major bleeding rates were significantly increased in the pre-treatment group at 7 days. Since then, there has been an extensive discussion and the topic remains controversial. In the PCI-CURE trial, clopidogrel pre-treatment compared to placebo, showed a reduction in cardiovascular death or MI. In addition, a subgroup analysis of the PLATO trial demonstrated equal benefit of DAPT with ticagrelor in patients intended for non-invasive treatment with NSTE-ACS. Currently, no guideline recommendations on pre-treatment are provided. Based on current available literature it can only be concluded that pre-treatment with prasugrel is contraindicated and for clopidogrel and ticagrelor only data from (underpowered) subgroup analysis are available. In NSTE-ACS patients with a conservative management, preferably ticagrelor, is recommended as soon as the diagnosis has been confirmed, of course only when there are no contraindications.

A specific, and increasing, subgroup of patients are the elderly. In the ACCOAST trial the increased bleeding risk with prasugrel was also present in the subgroup of patients aged >75 years. In the PCI-CURE trial subgroup of 1,042 patients aged ≥65 years, no significant benefit of clopidogrel pre-treatment compared with placebo was observed [30]. In the PLATO trial, patients ≥65 years demonstrated a superiority of ticagrelor compared with clopidogrel. However, in patients ≥75 years this superiority was no longer statistically significant [26]. On the other hand, ticagrelor was not associated with an increased bleeding risk compared with clopidogrel in this specific population. In conclusion, in elderly patients with a high bleeding risk it seems more reasonable to postpone the administration of a P2Y12 inhibitor until after angiography.

PRECISE-DAPT demonstrated improved integrated discrimination and reclassification performance as compared with other bleedings scores [31]. The PRECISE-DAPT can be used as an algorithm at the moment of hospital discharge to identify patients with an increased bleeding risk in whom duration of DAPT should be shortened. In addition, a DAPT score was developed to identify patients who might benefit of prolonged DAPT after 1 year of event-free treatment [26, 32, 33]. The duration of DAPT in patients with NSTE-ACS is extensively discussed elsewhere in this issue of the Netherlands Heart Journal [DOI].

### Anticoagulation

The combination of anticoagulation and platelet inhibitors is proven to be more effective than either treatment alone in reducing ischaemic events in NSTE-ACS patients [34].

#### Unfractionated heparin (UFH)

In patients with NSTE-ACS the use of UFH as an anticoagulant is still daily practice in a number of countries, however, there is evidence for an increased bleeding risk compared with other anticoagulants [35]. If UFH is used during PCI, it can be given under activated clotting time (ACT) guidance (250–350 s) or in a weight-adjusted manner (70–100 IU/kg). No dose adjustment to renal function is recommended.

#### Low-molecular-weight heparin (LMWH)

The most widely used LMWH in NSTE-ACS is enoxaparin at a dose of 1 mg/kg twice daily in patients with normal renal function. It has a more predictable dose-effect relationship than UFH. However, the treatment regimen in the period before, during and after the PCI can be confusing, as this depends on the last time of administration. Meta-analysis testing enoxaparin versus UFH in ACS demonstrated favourable safety and efficacy profiles of enoxaparin during, mainly primary, PCI [35]. Prior studies in NSTE-ACS patients treated conservatively demonstrated that enoxaparin was also superior to UFH [36].

#### Fondaparinux

The selective factor Xa inhibitor fondaparinux is considered to have the most favourable efficacy and safety profiles and is recommend as first-choice anticoagulation in NSTE-ACS, except for patients who are scheduled for immediate coronary angiography. Once-daily dosing is sufficient, but due to its renal elimination, it is contraindicated if eGFR <20 mL/min/1.73 m^2^. The OASIS-5 (Organization to Assess Strategies in Acute Ischaemic Syndromes 5) study tested enoxaparin versus fondaparinux and observed an equal efficacy, but a more favourable safety profile of fondaparinux [37]. We should point out that fondaparinux is associated with an increased risk of catheter thrombosis which can be prevented by an additional weight-adjusted bolus of UFH at the time of PCI.

#### Bivalirudin

The direct thrombin inhibitor bivalirudin has a more predictable anticoagulant effect than UFH. Bivalirudin has been tested against UFH in several trials with several different treatment regimens, including the ACUITY and ISAR-REACT 3 and 4 (Intracoronary Stenting and Anti-thrombotic Regimen—Rapid Early Action for Coronary Treatment) trials. In NSTE-ACS patients undergoing PCI bivalirudin has a comparable efficacy but is safer than UFH. It has a Class IA recommendation in the ESC NSTE guidelines, but still fondaparinux has the advantage of parenteral administration [6].

#### Specific comments on the Dutch health care system

In the Dutch health care system, the majority of patients admitted with NSTE-ACS are pre-treated with dual antiplatelet inhibition. The most important reason for this regimen is the fact that due to the current health care system the choice for conservative or invasive management in a significant part of the NSTE-ACS population is made during the first 48 h of admission and not directly at presentation. Therefore, treatment with a single platelet inhibitor during this period, could result in “undertreatment” of a significant part of this population. The Dutch ACS working group has therefore suggested pre-treatment of NSTE-ACS patients with low to moderate bleeding risk and high probability of PCI. In contrast, in patients with known coronary anatomy or clinical evidence of 3‑vessel disease/left main involvement (high likelihood of CABG), a choice for single antiplatelet therapy can be made.

## Invasive coronary angiography

### Routine versus selective invasive approach

In current Dutch practice, most patients with NSTE-ECS undergo invasive coronary angiography. The obtained information on coronary anatomy and disease is used for further decision making on the treatment regimen (pharmacological, PCI or CABG). Multiple studies and several meta-analyses have demonstrated that, compared with a selective invasive strategy, a routine invasive strategy in NSTE-ACS patients improves clinical outcomes and reduce recurrent ACS episodes, rehospitalisation and revascularisation [38–40].

### Timing of invasive strategy

In the ESC guidelines on management of NSTE-ACS, risk criteria are provided to support the clinical decision making on the timing of the invasive strategy. The very-high-risk criteria are: 1) haemodynamic instability or cardiogenic shock, 2) recurrent or ongoing chest pain refractory to medical treatment, 3) life-threatening arrhythmias or cardiac arrest, 4) mechanical complications of MI, 5) acute heart failure, and 6) recurrent dynamic ST-T wave changes, particularly with intermittent ST elevation. The three high-risk criteria are: 1) rise or fall in cardiac troponin compatible with MI, 2) dynamic ST-wave or T‑wave changes (symptomatic or silent), and 3) GRACE score >140. The seven intermediate-risk criteria are: diabetes mellitus, renal insufficiency (eGFR <60 mL/min/1.73 m^2^), LVEF <40% or congestive heart failure, early post-infarction angina, prior PCI, prior CABG, and GRACE risk score >109 or <140. Finally, the presence of any characteristics not mentioned in the prior criteria is mentioned as low-risk criteria [6].

#### Immediate invasive strategy

Performing a coronary angiography within 2 h is recommended in NSTE-ACS patients with at least one very-high-risk criterium as mentioned above. As most randomised trials have excluded these patients, it is recommended to treat this category of patients according to STEMI patients.

#### Early invasive or delayed invasive strategy

The ESC guidelines for NSTE-ACS recommend an early invasive approach (<24 h) in patients with at least one high-risk criterion [6]. This recommendation also includes timely transfer of patients admitted to hospitals without on-site catheterisation facilities. Importantly, this is announced as a ‘performance measure in NSTE-ACS’. These recommendations are based on 2 meta-analyses including in total seven RCTs in 5,370 NSTE-ACS patients and four observational studies including 77,499 patients, comparing early and delayed invasive strategy [41]. Remarkably, the only difference in favour of an early invasive strategy was a significant lower risk of refractory ischaemia, but not in in death or major bleeding event. Sub-analysis of two trials has provided some evidence in favour of an early invasive strategy only in patients with a high-risk profile. The TIMACS (Timing of Intervention in Acute Coronary Syndromes) trial demonstrated no difference between early and delayed invasive strategy in the complete study population. Only in a pre-specified sub-analysis of high-risk patients (one third of patients with a GRACE risk score >140) did an early invasive strategy lower the risk of death, MI or stroke at six months. However, the study was not powered for this subgroup analysis [42]. Of note, sub-analysis from a negative trial can at best be generating hypothesis but cannot really contribute to the implementation of new guidelines. In addition, in a post hoc analysis of the ACUITY trial, delayed invasive strategy in NSTE-ACS patients with high-risk criteria was an independent predictor of 30-day and 1‑year mortality, however, again the trial was not powered for this sub-analysis [43].

#### Specific comments on the Dutch health care system

In the Dutch health care system, the recommendations on invasive strategies in NSTE-ACS patients are not completely adopted, as earlier described in the consensus document of the ‘working group on ACS’ of the Dutch society of cardiology (NVVC) [44]. The most important reason is the lack of scientific evidence on a benefit of an early invasive versus a delayed invasive strategy. As described above, only sub-analysis of 2 RCTs demonstrated a possible benefit, however, as these are not adequately powered, these results can only be interpreted as hypothesis-generating results. In addition, in the Dutch health care system, there are many non-PCI hospitals with the presence of a cardiac care unit and the capability of performing coronary angiography during daytime only. Patients admitted to these hospitals with a NSTE-ACS are pharmacologically treated according the current ESC guidelines, and, if admitted during the weekend, coronary angiography is performed on the first day after the weekend. With this current practice, there are only rarely examples of calamities. Two important factors are the fundament for the adequate function of the current Dutch system; first, in unstable patients or patients with very-high-risk criteria the ambulance staff already decides to present the patient to a PCI centre, and second, if a prior stable NSTE-ACS patient who has been admitted to a non-PCI centre develops very-high-risk criteria, an immediate transfer to a PCI centre is always possible with very short time delays. The latter is due to the short travel distances between the hospitals and the adequately functioning ambulance services.

In the current NSTE-ACS guidelines the rise and fall of troponin is considered a high-risk criterium, and therefore should lead to same day transfer to a hospital with on-site catheterisation facilities. We would like to point out that this group of patients represents the majority of the NSTE-ACS population which is why, in our opinion, this criterium should not be applied directly to the Dutch system. Instead a rise in troponin levels should be assessed in relation to the patient’s clinical presentation as the current high-sensitive troponin assays very often detect a rise in troponin levels in cases with a non-coronary aetiology, i. e. pericarditis, myocarditis, pulmonary pathology, aortic pathology, and type 2 myocardial infarction. Most of these patients can be treated locally in a non-PCI centre. Importantly, the current standard of care in these patients has turned out to be safe and effective in daily care, therefore, in our opinion, same day transfer is not mandatory in most of these patients.

In NSTE-ACS patients admitted to a non-PCI centre, coronary angiography is performed locally and thereafter data are discussed in dedicated heart teams in centres with on-site cardiac surgery. Based on the decided treatment regimen, patients are transferred to the treating hospitals if necessary. Medical treatment is prescribed in keeping with existing guidelines during the waiting period for coronary angiography and DAPT is initiated based on the arguments described above.

## Conclusions

In the current NSTE-ACS ESC guidelines the importance of risk assessment is emphasised. Regarding ischaemic risk assessment, the GRACE 2.0 risk score seems most applicable. Importantly the ischaemic and bleeding risk scores have the major limitation of not being prospectively validated, and therefore there is a clear need for prospective testing of these risk scores in randomised clinical trials in NSTE-ACS patients. Depending on adequately validated risk scores, decision making regarding antithrombotic and anticoagulation therapy could be more tailored to the patient. Furthermore, especially in the Dutch health care system, the decision for early or delayed invasive strategies should be patient tailored, and not solely be based on a short list of high-risk criteria provided by non-prospective validation cohorts.
